# Epidemiology of *Salmonella* sp. in California cull dairy cattle: prevalence of fecal shedding and diagnostic accuracy of pooled enriched broth culture of fecal samples

**DOI:** 10.7717/peerj.2386

**Published:** 2016-08-30

**Authors:** Omran A. Abu Aboud, John M. Adaska, Deniece R. Williams, Paul V. Rossitto, John D. Champagne, Terry W. Lehenbauer, Robert Atwill, Xunde Li, Sharif S. Aly

**Affiliations:** 1 School of Veterinary Medicine, Veterinary Medicine Teaching and Research Center, University of California, Davis, Tulare, California, United States; 2 California Animal Health and Food Safety Laboratory, Tulare, California, United States; 3 Department of Population Health and Reproduction, University of California, Davis, California, United States; 4 Western Institute for Food Safety and Security, University of California, Davis, California, United States

**Keywords:** *Salmonella*, Cull dairy cows, Prevalence, Fecal pooling, Enriched broth culture, Sensitivity, Specificity, California

## Abstract

**Background:**

The primary objective of this cross-sectional study was to estimate the crude, seasonal and cull-reason stratified prevalence of *Salmonella* fecal shedding in cull dairy cattle on seven California dairies. A secondary objective was to estimate and compare the relative sensitivity (Se) and specificity (Sp) for pools of 5 and 10 enriched broth cultures of fecal samples for *Salmonella* sp. detection.

**Methods:**

Seven dairy farms located in the San Joaquin Valley of California were identified and enrolled in the study as a convenience sample. Cull cows were identified for fecal sampling once during each season between 2014 and 2015, specifically during spring, summer, fall, and winter, and 10 cows were randomly selected for fecal sampling at the day of their sale. In addition, study personnel completed a survey based on responses of the herd manager to questions related to the previous four month’s herd management. Fecal samples were frozen until testing for *Salmonella*. After overnight enrichment in liquid broth, pools of enrichment broth (EBP) were created for 5 and 10 samples. All individual and pooled broths were cultured on selective media with putative *Salmonella* colonies confirmed by biochemical testing before being serogrouped and serotyped.

**Results:**

A total of 249 cull cows were enrolled into the study and their fecal samples tested for *Salmonella*. The survey-weighted period prevalence of fecal shedding of all *Salmonella* sp. in the cull cow samples across all study herds and the entire study period was 3.42% (N = 249; SE 1.07). The within herd prevalence of *Salmonella* shed in feces did not differ over the four study seasons (P = 0.074). The Se of culture of EBP of five samples was 62.5% (SE = 17.12), which was not statistically different from the Se of culture of EBP of 10 (37.5%, SE = 17.12, P = 0.48). The Sp of culture of EBP of five samples was 95.24% (SE = 3.29) and for pools of 10 samples was 100.00% (SE = 0). There was no statistical difference between the culture relative specificities of EBP of 5 and 10 (P > 0.99).

**Discussion:**

Our study showed a numerically higher prevalence of *Salmonella* shedding in the summer, although the results were not significant, most likely due to a lack of power from the small sample size. A higher prevalence in summer months may be related to heat stress. To detect *Salmonella*, investigators may expect a 62.5% sensitivity for culture of EBP of five, relative to individual fecal sample enrichment and culture. In contrast, culture of EBP of 10 samples resulted in a numerically lower Se. Culture of EBP of size 5 or 10 samples, given similar prevalence and limit of detection, can be expected to yield specificities of 95 and 100%, respectively.

## Introduction

Over one million cases of non-typhoidal *Salmonella* infections are estimated to occur each year in the United States, of which 94% are foodborne (Scallan et al., 2011). Additionally, non-typhoidal *Salmonella* infections are estimated to be the leading cause of foodborne hospitalizations and deaths in the US (Scallan et al., 2011). In a multi-site US study, consumption of undercooked ground beef was shown to be the strongest risk factor for infection with multidrug resistant *Salmonella enterica* serotype Newport (Varma et al., 2006). Cull dairy cows account for approximately 18% of ground beef production in the United States (NAHMS, 1996). A previous study that collected cecal-colon samples from cull dairy cattle at a slaughterhouse reported prevalence of *Salmonella* sp. between 9.6 and 93.0% in the Western US, depending on the season and day of the week that the samples were collected (Troutt et al., 2001). Such a wide prevalence range may be due to trends in fecal shedding or risk factors that may be significantly associated with shedding and that may differ between dairies. However, this study did not collect information on the dairies from which the study cows originated (Troutt et al., 2001). Hence, the goal of the current study was to report on the prevalence of *Salmonella* in cull dairy cattle destined for market within 24 h by season and reason for culling.

Traditionally, the prevalence of *Salmonella* has been determined using individual fecal sample culture methods. Singer et al. (2006) showed that the use of PCR on pools of five fecal samples can improve the speed and efficiency of detecting *Salmonella* in dairy cattle feces. However, the study was not specific to cull dairy cattle. Other limitations included non-random sampling of cattle, lack of serotype information for *Salmonella* isolates and the high cost of PCR despite cost savings from pooling (Singer et al., 2006). Alternatively, culture of pooled fecal samples from cull dairy cattle may provide cost-savings compared to PCR, provided the sensitivity of detection remains acceptable after pooling.

Pools of 5, 10 or more fecal samples have previously been investigated for *Mycobacterium avium subsp. paratuberculosis* (Aly et al., 2012). Due to the dilution effect, both the prevalence of shedding in the cohort of cows sampled and number of positive samples within a pool have an effect on the pooling sensitivity (Rours et al., 2005; Singer et al., 2006; Muniesa et al., 2014). Often samples submitted to a diagnostic laboratory are pooled in the order they are presented or by the groups of animals sampled, which may result in pools with variable percent of positive samples, and, therefore, variable concentrations of the target analyte, which may affect sensitivity of culture, if the final concentration of viable *Salmonella* bacteria is less than the detection threshold. One solution to this problem is random ordering of individual samples that go into any pool, which may result in comparable analyte concentrations across positive pools.

Turnaround time for final results may be increased with pooling due to the pending testing of individual samples once a positive pool is identified. Final results are further delayed with *Salmonella* detection using culture of pooled samples due to a common and necessary enrichment step. An efficient approach to testing pooled samples for *Salmonella* is testing enriched broth pools (EBP) created from the individual broths instead of pooling individual fecal samples, as previously described (Singer et al., 2006). The importance of culturing EBP for *Salmonella* sp. may depend on the number of samples pooled. Once an optimum pool size is determined, the diagnostic accuracy of pooling should be estimated to allow for cost-effectiveness studies that can provide a road map for testing whole herds. Such an approach has been previously proposed for diseases such as mastitis on large dairy herds (Murai et al., 2014). Hence, a second goal of the current study was to estimate the relative sensitivity (Se) and specificity (Sp) to detect *Salmonella* using individual sample culture results as the reference status (Muniesa et al., 2014). The primary objective of this cross-sectional study was to estimate the crude, seasonal and cull-reason stratified prevalence of *Salmonella* fecal shedding in cull dairy cattle on seven California dairies. A secondary objective was to estimate and compare the Se and Sp for pools of 5 and 10 enriched broth cultures of fecal samples for *Salmonella* sp. detection. Results of the current study will guide individuals surveying dairy cattle for *Salmonella* shedding using pooled fecal samples and interpretation of their culture results.

## Materials and Methods

### Farms and sampling

The study was approved by the University of California, Davis Institutional Animal Care and Use Committee (protocol number 18019). Seven dairy farms located in the San Joaquin Valley of California were identified and enrolled in the study as a convenience sample. A sample size of 246 cull dairy cows was required to produce a two-sided 95% confidence interval with a width of 10% assuming a prevalence of 50%, the latter maximizes sample size required when the true prevalence is not known (Newcombe, 1998, Fleiss, Levin & Paik, 2003). Cull cows were identified for fecal sampling once during each season between 2014 and 2015, specifically during spring (April–June), summer (July–September), fall, (October–December) and winter (January–March). The choice of week to sample cull cows during any of the four seasons was also by convenience. From the list of cows selected by the dairy farms for culling and sale for beef, 10 cows were randomly selected for fecal sampling at the day of their sale using a random number generator from the available cow sale list. Random numbers were prepared specific to the total possible number of cows being presented for sampling with a specific list for each of the sampling frame 11–20, 21–30, 31–40, 41–50, 51–60, 61–70, 71–80, 81–90 and 91–100 cows. If a producer had less than 11 cows available for sale on a given sampling day, then all cows were sampled at that time. Based on the information provided by the owner or herd manager at the day of culling, the respective list of random numbers was used to identify cull cows for fecal sampling. Fecal samples were collected manually using individual disposable sleeves from the rectum of the randomly selected cows and transported to the Dairy Epidemiology Lab (Aly Lab) on wet ice for processing within 2–6 h of sampling. Fecal samples were stored in polypropylene tubes at −80 °C until completion of sampling in 2015, at which time all the samples were cultured for *Salmonella*.

### Relational database

On the day of sample collection, study personnel completed a survey based on responses of the herd manager to questions related to the previous four month’s herd management including herd size, breed distribution, milk production, culling rate, number of times cows were culled per month, percent of cull cows sold for beef (compared to dairy), feeding a special fattening diet for cull cows, use of a specific pen for cull cows, rate of manure removal from pens, percent of cull cows condemned and reason for condemnation. Herd managers were also asked questions about the previous four months percent of cull cows that received injectable medical treatments, percent of culled cows that received injectable treatments three weeks prior to culling, personnel allowed to administer drugs, drug residue avoidance (use of specific drugs, observing withdrawal time, testing milk and or urine prior to culling, or other actions), how were withdrawal periods tracked, use of a drug inventory system and extralabel drug use (familiarity and frequency). In addition, a backup of the herd’s Dairy Herd Improvement software file was obtained within a week of the visit to extract cull cows’ milk production and health events data. Data from all sources were housed and linked in a relational database using dairy and cow identification, and date of sampling (Microsoft Office, Access 2013, Redmond, WA).

### Bacteriological culture

Frozen fecal samples were thawed at room temperature and, for each sample, 1 gram of feces mixed with 9 ml of Selenite Broth (Vet Med Biological Media Services, Davis, CA, USA) was incubated for 18–20 h at 37 °C. After overnight incubation EBP of 5 and 10 samples were created as described below and were plated onto solid media at the same time the broth from individual fecal samples were plated to maintain blinding of culture results of individual samples when creating pools. A cotton swab from each Selenite broth culture of individual samples and pools of 5 and 10 was used to streak for isolation on Hektoen Enteric (HE) Agar containing 10 μg/L Novobiocin and incubated for another 18–24 h at 37 °C. Five distinct and spatially isolated, putative *Salmonella* colonies, greenish blue with black centers, were selected from each positive HE plate and streaked on Sheep Blood Agar for further testing. For biochemical testing, the selected colonies were inoculated into Urea agar slants and Triple Sugar Iron agar slants. Colonies were designated as suspect *Salmonella* if they were urease negative, dextrose fermenting, and produced H_2_S. Suspect *Salmonella* colonies, from the biochemical testing, were confirmed using commercial polyvalent A1 and Vi antisera (DIFCO, Becton Dickinson Co., Sparks, MD) following the manufacturer’s instructions. Subsequently, confirmed colonies were tested for the individual serogroups associated with the most common bovine *Salmonella* isolates of B, C1, C2, D1, and E. For each group identified, a single colony was submitted to the California Animal Health and Food Safety Lab (CAHFS) for serotype determination.

### Preparation of pools of 5 and 10 enriched broths

EBP were created using the overnight incubated Selenite broths. To reduce the chance of multiple *Salmonella* positive broths within a single EBP, 0.5 ml of the vortexed Selenite broth from individual cow fecal samples, from the same season and dairy, were randomly selected for preparation of each EBP of five samples. An EBP of 10 samples was then created by pooling 0.5 ml from each of the 10 Selenite broths representing each season and dairy.

### Statistical analysis

Characteristics of the study herds over the study period, including milking herd size, predominant breed(s), rolling herd average and cull cow management, were summarized.

#### Salmonella shedding prevalence

The survey-weighted prevalence of *Salmonella* shedding in the population of cull dairy cattle was estimated using a stratified random sample of cull cows, within the strata dairy and season. Weights for each of the 10 randomly selected cows were assigned based on the total number of cows presented for culling on sampling day. The test of independence using Pearson’s chi-square statistic was computed to compare the survey-weighted prevalence of *Salmonella* sp. over the study seasons, both overall and for each dairy. The culling reason-specific prevalences were estimated as the survey-weighted proportion of *Salmonella* positive fecal samples and compared using the Pearson’s chi-square statistic.

#### Estimation of relative sensitivity and specificity of pooling

The Se of pooling of EBP was estimated as:

$${{Number\;of\;culture\;positive\;EBP\;containing\;at\;least\;one\;positive\;individual\;broth\;} \over {Predicted\;number\;of\;culture\;positive\;EBP\;based\;on\;positive\;individual\;broth\;}}$$

where positive is culture positive for a known serogroup of *Salmonella* (Muñoz-Zanzi et al., 2006; Muniesa et al., 2014).

McNemar’s test was used to test the hypothesis that the sensitivity of EBP of 5 was not equal to EBP of 10. Similarly, the Sp of pooling of EBP was estimated as:

$${{Number\;of\;culture\;negative\;EBP\;containing\;all\;negative\;individual\;broth\;}\over{Predicted\;number\;of\;culture\;negative\;EBP\;based\;on\;negative\;individual\;broth\;}}$$

and compared between EBP of 5 and 10 samples (Muñoz-Zanzi et al., 2006). A 5% level of significance was used for statistical comparisons. All data analysis was performed using Stata 14.0 (College Station, TX).

## Results

### Descriptive statistics

#### Study herd characteristics and management

Table 1 summarizes the characteristics of the study herds and management practices related to culling cows during each of the study’s seasons. None of the study dairies fed a special diet for cows identified for future culling, or housed cull cows in a designated cull cow pen prior to culling. All the study dairies used recycled lagoon water to flush manure from pens. Producers on all the study dairies reported that cows culled were all sold for beef, with the exception of herd 3 where, although all cows culled on sampling days were sold for beef, on non-sampling days cows may have been sold for non-beef purposes (66% in spring, 0% in summer, 66% in fall, and 70% in winter; mean over study period 57.9%). With the exception of herd 2, all the study herds had at least one cow condemned at an abattoir during the study period. Herds 1 and 3 had < 1% of their culled cows condemned while herds 4–7 had < 3% and their managers either did not list a reason, or, indicated that the reason for condemnation was unknown.

**Table 1:** Characteristics of seven California dairy herds enrolled in a cross-sectional study to survey for *Salmonella* sp. fecal shedding in a random sample of cull dairy cows.

Herd	Mean milking herd size (SE)	RHA,^a^ Kg (SE)	Herd breed^b^ distribution, (%)	Collected samples breed distribution, (%)	Herd percent culled per month, % (SE)	Culling times per month	Facility design^c^
1	3,763 (180)	10,768 (451)	H (45%), J (55%)	H (63%), J (37%)	2.66 (0.04)	1 to 2	FS
2	2,810 (29)	11,546 (111)	H (100%)	H (100%)	3.13 (0.17)	4 to 5	FS
3	3,050 (102)	8,217 (440)	J (100%)	J (100%)	3.48 (0.2)	1 to 4	DL
4	5,600 (141)	13,467 (109)	H (95%), J (5%)	H (100%)	2.83 (0.32)	1 to 4	FS
5	2,633 (113)	10,878 (26)	H (100%)	H (100%)	2.37 (0.01)	1	DL
6	839 (39)	12,500 (228)	H (100%)	H (100%)	8.82 (0.29)	1	FS
7	1,606 (37)	14,559 (367)	H (100%)	H (100%)	2.4 (0.07)	1	DL
All	3,059 (1,386)	10,297 (202)			3.27 (0.13)		

**Notes:**

a Rolling herd average defined as the mean milk produced per milking cow in the herd in 365 days.

b (H), Holstein and (J), Jersey breeds.

c Facility design: (FS), freestall and (DL), drylot.

At each of the four season’s surveys, herd 1’s manager reported 0% of cull cows received antibiotic injections in the three week period prior to culling. In contrast, managers of the remaining herds reported 1.99% (SE < 0.01) of cull cows received antibiotic injections in the three-week period prior to culling. On all study dairies, treatments were injected by a designated employee, and, to avoid drug residues, all except herd 3 reported limiting treatments to specific drugs. Only herd 6 reported testing cattle for antibiotic residues by submitting milk samples to the contracting creamery for antibiotic residue testing. In addition to the use of computerized software to track withdrawal periods, managers reported some use of chalk markings on cows (all herds), paper records (all except herd 4), memory (herd 1 only) and use of leg bands (herds 2, 4 and 5). A drug inventory with names and quantities of drugs on the dairy was kept only on herd 6. In addition, managers of all the study herds reported recording date of treatment, herds 4, 5 and 7 reported recording dose of drug and route of administration while herd 2 recorded only dose of drug. Managers of the study herds were all familiar with extralabel drugs. Herd 6 and 7 managers reported no extralabel drug use, herd 1 reported variable frequency of extralabel drug use while the remaining herds reported a mean of four times/month (SE = 0.01). Only herds 2, 4 and 7 used a *Salmonella* vaccine as part of their vaccination program.

#### Period prevalence of fecal shedding of *Salmonella*

A total of 814 cows were presented for culling at sampling days on the study herds during the study period. Fecal samples were collected from 10 cull cows on each study dairy at each of the seasons with the exception of herd 6, which was only sampled in spring and summer due to cull cows being sold prior to coordinating with the study authors and only had nine cows sampled during the spring. Hence, the 50th EBP of five was made of four enriched broth samples, similarly, the 25th EBP of 10 was made of nine. In addition, 10 samples collected from herd 1 at spring of 2014 were excluded from the study as they were not collected from a random sample of the cull cows sold on sampling day. Hence, a total of 249 cull cows were enrolled into the study and their fecal samples tested for *Salmonella*. Data records for two of the enrolled cows were missing breed information.

The period prevalence of fecal shedding of *Salmonella* of all serotypes in the cull cow samples from all the study herds and over the entire study period was 3.42% (N = 249; SE 1.07; 95% CI 1.24, 9.02). The prevalence of *Salmonella* shed in feces of sampled cows did not differ over the four study seasons (P = 0.074; Table 2). The frequency of *Salmonella* isolates shed in feces of the study’s cull cows, by serogroup and serotype, are summarized in Table 3. Cows were culled due to a variety of cull reasons, primarily low milk production followed by poor reproductive outcomes (Table 4). The remaining culling reasons included lameness, post-calving reproductive pathology, mastitis, metabolic disease, other, or unknown reasons. Other reasons included unknown illness, gastrointestinal disorder, poor udder conformation, undiagnosed fever, pneumonia, or eye disease. Herd managers reported at least one culling reason for 244 sampled cows, culling reason for the remaining five (2.0%) was either not reported (two cows) or missing (three cows) due to a mismatch in cow identification number and survey records. All five cows with no culling reason were *Salmonella* negative. Of the cows with a known reason for culling, 104 (41.8%), 122 (49.0%), 14 (5.6%) and 3 (1.2%) had 1, 2, 3 and 4 culling reasons, respectively.

**Table 2:** Survey-weighted prevalence, by herd and season, of *Salmonella* sp. shed in feces of 249 culled cows sampled on seven California dairies.

Herd	Prevalence % (SE), n^a^, N^b^					P value	Serotype
	Spring^c^	Summer	Fall	Winter	Overall		
1	–^d^	10.0 (7.64), 10,24	0 (−), 10, 31	0 (−), 10, 67	1.97 (1.50), 30, 122	0.538	*S.* Typhimuium
2	0 (−), 10, 13	10.0 (6.88), 10, 19	0 (−), 10, 24	0 (−), 10, 28	2.26 (1.56), 40, 84	0.80	*S. I1 4, 5, 12: i-*
3	0 (−), 10, 26	10.0 (8.23), 10, 31	0 (−), 10, 41	20.0 (10.88), 10, 30	7.11 (3.24), 40, 128	0.124	*S.* Montevideo
4	0 (−), 10, 61	10.0 (9.44), 10, 65	0 (−), 10, 71	0 (−), 10, 52	2.61 (2.43), 40, 249	0.182	*S.* Bardo
5	0 (−), 10, 17	0 (−), 10, 20	10.0 (8.58), 10, 38	0 (−), 10, 36	3.42 (2.94), 40, 111	0.209	*Unidentified Salmonella*^e^
6	10.0 (3.02), 10, 11	0 (−), 9, 9	–	–	5.50 (1.66), 19, 20	0.424	*S.* rough type O
7	0 (−), 10, 16	10.0 (8.16), 10, 30	0 (−), 10, 31	0 (−), 10, 23	3.00 (2.45), 40, 100	0.164	*S.* Montevideo
All	0.76 (0.23), 60,144	8.54 (3.75), 69, 198	1.61 (1.38), 60, 236	2.54 (1.38), 60, 236	3.42 (1.07), 249, 814	0.074	

**Notes:**

a Number of dairy cows randomly selected for fecal sampling from the list of cows identified for culling at sampling day.

b Number of dairy cows identified for culling at sampling day.

c Study year and seasons included summer (July 1–September 30, 2014), fall (October 1–December 31, 2014), winter (January 1–March 31, 2015) and spring (April 1–June 30, 2015).

d No samples cultured due to non-random selection of culled cows.

e Unidentified polyvalent positive *Salmonella* sp.

**Table 3:** Frequency and survey-weighted prevalence of *Salmonella* sp. isolates by serogroup and serotype classification after culture of fecal samples from 249 culled cows on seven California dairies sampled over a year.

Serogroup	Serotype(s)	Number of isolates	Prevalence (%)	SE	95% CI	
					Lower	Upper
B	All	2	0.53	0.28	0.10	2.76
	*S.* Typhimuium	1	0.29	0.23	0.03	3.27
	*S. I 1,4,5,12:i:-*	1	0.23	0.16	0.03	2.06
C1	*S.* Montevideo	4	1.49	0.59	0.42	5.17
C2	*S.* Bardo	1	0.80	0.74	0.04	13.69
D	*S.* rough type O	1	0.14	0.04	0.05	0.35
Polyvalent	*Salmonella* sp.^a^	1	0.47	0.40	0.03	6.80

**Note:**

a Unidentified polyvalent positive *Salmonella* sp.

**Table 4:** Survey-weighted proportion for culling reasons for 249 cows on seven California dairies surveyed over a course of a year.

Culling reason	Season^a^										P value
	Spring (N = 60)		Summer (N = 69)		Fall (N = 60)		Winter (N = 60)		Overall (N = 249)^b^		
	N	% (SE)	N	% (SE)	N	% (SE)	N	% (SE)	N	% (SE)	
Low milk production	36	45.98% (5.89)	61	86.92% (4.64)	45	77.42% (4.95)	48	83.39% (3.93)	190	75.90% (2.40)	0.003
Poor reproduction	36	54.03% (6.88)	31	35.61% (5.43)	26	43.52% (6.16)	24	44.87% (5.75)	117	43.85.0% (3.04)	0.336
Lameness	5	11.60% (5.26)	7	9.49% (2.70)	5	5.38% (1.67)	5	6.10% (2.31)	22	7.69% (1.41)	0.441
Mastitis	11	12.22% (2.18)	5	8.03% (3.60)	4	5.68% (2.44)	4	6.10% (2.69)	24	7.53% (1.42)	0.479
Metabolic disease	0	–	2	1.92% (0.88)	3	3.05% (1.19)	0	–	5	1.35% (0.41)	0.044
Post-calving reproductive issues	4	13.82% (5.96)	4	6.1% (2.03)	4	8.94% (4.11)	0	–	12	6.51% (1.67)	0.094
Other^c^	5	18.06% (6.36)	8	12.93% (3.9)	8	15.59% (4.84)	11	12.25% (2.47)	32	14.41% (2.16)	0.785

**Notes:**

a Study year and seasons included summer (July 1–September 30, 2014), fall (October 1–December 31, 2014), winter (January 1–March 31, 2015) and spring (April 1–June 30, 2015).

b Totals and percents do not add up to 249 or 100%, respectively, due to multiple cull reasons.

c The category labeled other reasons included the following conditions: unknown illness, gastrointestinal disorder, poor udder confirmation, undiagnosed fever, pneumonia or eye disease.

Of the cull cows shedding *Salmonella* in their feces, both the cow shedding *Salmonella* rough type O and the cow shedding *S.* I 1,4,5,12:i- were culled due to low milk production and poor reproductive outcomes. The cow that shed *S.* Typhimurium was culled due to low milk production and lameness. The four cows that shed *S.* Montevideo were culled for low milk production, poor reproduction or miscellaneous reasons. The cow shedding *S.* Bardo was culled due to miscellaneous reasons, similar to the cow that shed an unidentified *Salmonella* sp. except that the latter also had low milk production and poor reproduction.

#### Relative sensitivity and specificity of EBP culture

A total of 50 EBP of size five were generated, seven EBP each contained one *Salmonella* positive enriched broth of an individual cow fecal sample and one EBP had two. Of the eight predicted culture positive EBP, five cultured positive. Hence, the Se of culture of EBP of five samples was 62.5% (SE = 17.12, 95% CI 28.95, 96.05). Similarly 25 EBP of 10 samples were generated, seven EBP each contained one *Salmonella* positive enriched broth of an individual cow fecal sample and one EBP had two. Of the eight predicted culture positive EBP, three cultured positive. Hence, the Se of culture of EBP of 10 samples was 37.5% (SE = 17.12; 95% CI 3.95, 71.05). There was no statistical difference between the culture relative sensitivities of EBP of 5 and 10 (P = 0.48). Each of the false negative EBP of sizes 5 and 10 contained a single *Salmonella* positive enriched broth of an individual cow fecal sample.

Of the 42 EBP of size five predicted to be culture negative due to containing no positive enriched broth from individual cows, 40 cultured negative. Hence, the Sp of culture of EBP of size five was 95.24% (SE = 3.29, 95% CI 88.80, 100.00). The five individual constituents of one of the false positive EBP of size five tested negative and were combined with four negative individual samples to make the one EBP of size nine (herd 6) that also tested negative. The individual constituents that made up the second false positive EBP of five also tested negative and were combined with five additional individual samples that contained one positive sample to make an EBP of size 10 that tested positive. Of the 17 EBP of size 10 predicted to be culture negative due to containing no positive enriched broth from individual cows, all tested negative. Hence, the Sp of culture of EBP of size 10 was 100.00% (SE = 0; 95% CI 100.00, 100.00). There was no statistical difference between the culture relative specificities of EBP of 5 and 10 (P > 0.99).

## Discussion

The current study is the first to estimate the pre-harvest prevalence of fecal shedding of *Salmonella* sp. in cull dairy cattle on California dairies. Collecting fecal samples from a random sample of cull dairy cows from a convenience sample of seven large dairies in California, year round, provided a valid estimate for *Salmonella* shedding prevalence preharvest. The prevalence of *Salmonella* sp. in the feces of cull dairy cattle in the current study was lower than has been previously reported for dairy cattle in other studies across the U.S. or other states. Wells et al. (2001) using data collected from the NAHMS (1996) study sampled from February to July 1996, reported an overall prevalence of fecal shedding of *Salmonella* of 5.4% in milking cows and 18.1% in cows to be culled across U.S. dairies and 14.9% for cull dairy cows at markets across the U.S. In contrast to the current study, the Wells et al. (2001) study collected samples from both cows due for culling within the next seven days and milk cows within the herd. Additionally, the Wells et al. (2001) study only sampled small dairies (< 100 milking cows) once from late February–May 1996, but sampled large dairies (> 100 milking cows) once between late February and May 1996 and then two additional times between May and July 1996. The additional sampling of large dairies during the summer months may have biased the results to show a higher prevalence among the larger herds. Using a similar sampling scheme and data from the NAHMS 2007 study conducted between February and August 2007, Lombard et al. (2012) reported an overall fecal *Salmonella* prevalence in individual cows of 14% in dairies across the U.S., with a prevalence of 3.9% in the West region of the U.S. While this prevalence in the West region is similar to that reported in the current study, the lack of random sampling in the Lombard et al. (2012) study may have biased their results. Blau et al. (2005) used data from the NAHMS 2002 study, which included samples from milk cows over two years of age between March and September 2002, and reported a fecal *Salmonella* prevalence of 7.3% in dairies across the U.S. Possible reasons for the decreased prevalence of *Salmonella* in the current study, compared to previous reports, include use of a convenience sample of herds, differences in geographic regions and weather patterns at the time of sampling, differences in management practices between the study herds and differences in culturing methods used between the studies. In addition, the low prevalence of *Salmonella* in the study herds and specifically at the serotype level made it difficult to study the distribution of any specific serotype by cull reason. Furthermore, four of the nine *Salmonella* isolates were from a single serogroup (C1) and serotype (*S.* Montevideo), similar to a previous statewide study of calves raised on California dairies and calf ranches (Berge, Moore & Sischo, 2006).

*Salmonella* has been shown to be associated with season, with an increased prevalence in the summer months (Fossler et al., 2005; Edrington et al., 2008). Our study showed a numerically higher prevalence of *Salmonella* shedding in the summer, although the results were not significant, most likely due to a lack of power from the small sample size. A higher prevalence in summer months may be related to heat stress, although such an association may be confounded by cow and management related factors such as increased water/moisture in environment due to feed bunk sprinkler use to mitigate heat stress (Edrington et al., 2009). Nevertheless, the higher prevalence of *Salmonella* shedding in the summer is in agreement with the previous studies that showed an increased prevalence in the summer months (Wells et al., 2001; Blau et al., 2005; Lombard et al., 2012). However, none of these three studies sampled cows throughout the entire year as was done in the current study. These previous studies sampled cows from either late winter or spring to summer months. This may also explain the lower overall prevalence observed in the current study compared to the previous studies. It is interesting that the second highest prevalence of *Salmonella* shedding in this study occurred in the winter months (January–March) which may be due to higher moisture content in the dairy cow’s environment. Although, there was no statistically significant association between season and *Salmonella* fecal shedding prevalence in the current study, the absolute absence of *Salmonella* shedding (0% prevalence) year round with the exception of summer season, in four of the seven study herds may indicate a seasonal shedding pattern. Alternatively increased *Salmonella* transmission, infection and shedding in dairy cattle during periods of stress, such as hot summer months and cold moist winters, overcrowding or negative energy balance, should be investigated in future research. In addition, the role of antimicrobial drug use should be explored as a risk factor for *Salmonella* shed in dairy cattle feces (Warnick et al., 2003).

To detect *Salmonella* carriers or infected cattle, investigators may collect fecal samples from cows and culture EBP of five samples followed by culture of the respective individual broths that constitute positive pools to expect a 62.5% sensitivity, relative to individual fecal sample enrichment and culture. In contrast, culture of EBP of 10 samples resulted in a lower Se compared to that of pools of five samples and although the difference was not significant, the sample size for this comparison was small. Future research should be done using a larger sample size to confirm both the results for the sensitivities of pools of 5 and 10 EBP and the lack of difference between these two sensitivities. The use of EBP may decrease the turnaround time for final results, when culturing pooled samples, by avoiding enrichment of individual fecal samples a second time after positive fecal pools are identified. In the current study, overnight enriched broth from individual fecal samples was used to culture both EBP and individual samples. In laboratory applications, an aliquot of enriched broth from individual samples would be used to create EBP followed by another aliquot frozen for culture if the respective pool tested positive. The freeze-thaw of individual broth samples may negatively impact culture results for the individual samples and, as a result, the Se of pooling in this study may have been overestimated since individual samples were not frozen and then thawed once culture results from the pools were known.

Culture of EBP of size 5 or 10 samples, given similar prevalence and limit of detection, can be expected to yield relative specificities of 95 and 100% respectively. The current study results identified false negative and false positive EBP of size five. False negative pools may be explained by the dilution of the *Salmonella* colony forming units below the detectable concentration using culture. On the other hand, false positive pools can be explained by cross-contamination or imperfect sensitivity of the individual broth cultures leading to a false negative in at least one of the five individual samples in the pool. One of the two false positive EBP of size five shared constituent samples with a negative EBP of size nine that was made up of nine negative individual samples, which may support the possibility of cross-contamination. Another explanation would be dilution of a positive individual sample when making an EBP of size nine; however, given that all the individual samples that contributed to that pool were negative, this seems unlikely. Another explanation, although unlikely, is collection of an aliquot containing less than the minimum detectable *Salmonella* concentration, mix up of samples, or growth inhibition for reasons related to the medium or its incubation.

The current survey investigated antimicrobial drug use on the study herds, which revealed general awareness for antimicrobial stewardship as evident from lack of antibiotic treatments three weeks prior to culling, designating a specific employee for treatments, limiting treatments to specific drugs of suitable withdrawal periods and knowledge of extralabel drug use. Tracking withdrawal periods using different methods on the study dairies is a common practice. However, use of reproducible standardized records in combination with readily observable marking systems such as computer records and animal leg bands or markings may allow producers multiple levels of verification for drug residue status of cows prior to culling.

One of the limitations of the current study is the convenience sample of dairies enrolled in the study. Although the survey sampling approach stratified by dairy and season implied a random sample of dairies selected for cull cow sampling, the study herds were a convenience sample primarily based on the willingness of the producers to participate in the study. The latter raises the potential for bias towards producers who may not be representative of the remaining dairies in the San Joaquin Valley of California. Nevertheless, despite the use of a convenience sample of herds, this study included dairies that reflected the range of typical management practices and facility designs present throughout the valley. In comparison to an earlier survey that found that 50% of California dairies maintained a drug inventory, only 14.3% (1 of 7 herds) of the current study dairies did (Aly et al., 2014). In contrast, a larger proportion of the current study herd managers (100%) were familiar with extralabel drugs compared to the rest of the state (76%) (Aly et al., 2014). However, a similar proportion, 71.4% (5 of 7) of the current study dairies reported extralabel drug use compared to 64.4% across the state (Aly et al., 2014). Selection of sampling week within any season could have been random however this was not feasible given the variable culling practices that were affected by test-day milk production reports, market price and, more importantly, the producer’s schedule and willingness to inform and coordinate with our study team prior to culling. Hence, sampling cows at each culling on the study dairies may have resulted in different within-herd prevalence estimates of *Salmonella* shedding. In contrast, given that sampling cull cows from the study dairies occurred over several weeks within any entire season, the seasonal estimates are valid estimates of the seasonal fecal shedding of *Salmonella* in cull dairy cattle on the study dairies.

Due to the limitations of the current study and low prevalence of *Salmonella* shedding, it was not possible to investigate any causal associations between potential risk factors and shedding of *Salmonella* sp. in the cull dairy cattle. However, future research should be aimed at designing prospective, longitudinal studies to identify which risk factors contribute to the presence of *Salmonella* sp. in cull dairy cattle. Specific areas to be addressed include contamination of feed commodities, shedding of *Salmonella* sp. by wildlife, such as rodents and birds, and various management factors that might contribute to the seasonality of *Salmonella* sp. shedding.

## Conclusions

While *Salmonella* was present on all farms sampled in the current study, the overall prevalence in preharvest cull cows on a convenience sample of dairies in the San Joaquin Valley of California was lower than previously reported in dairy cattle across the US. Additionally, our study showed a numerically higher prevalence of *Salmonella* shedding in the summer compared to other seasons. To detect *Salmonella*, investigators may expect a 62.5% sensitivity for culture of EBP of five, relative to individual fecal sample enrichment and culture. In contrast, culture of EBP of 10 samples resulted in a lower Se. Culture of EBP of size 5 or 10 samples, given similar prevalence and limit of detection, can be expected to yield specificities of 95 and 100%, respectively.

## Additional Information and Declarations

### Data Deposition

The following information was supplied regarding data availability: The de-identified data was shared for peer review only as the dairy owners did not consent to publishing it alongside the article.
